# Analysis of the Coping Process among Visually Impaired Individuals, Using Interpretative Phenomenological Analysis (IPA)

**DOI:** 10.3390/ijerph17082819

**Published:** 2020-04-19

**Authors:** In Ok Sim

**Affiliations:** Red Cross College of Nursing, Chung-Ang University, Seoul 06974, Korea; hiraly@cau.ac.kr

**Keywords:** experience, coping, process, visual impairments, interpretative phenomenological analysis

## Abstract

There is a lack of research based on in-depth theoretical and scientific knowledge to understand the visually impaired, and there has been little effort in the application of strategies for early intervention to minimize the risk these people might encounter during development. This study used semi-structured interviews from eight persons with visual impairments who had various experiences of coping process. Three coping processes based on life experiences were identified: (1) self-awareness and adaptation process: “self-awareness of disability” and “adaptation to disability and the environment”; (2) facing the circumstance process: “the exposure to concealment and abuse,” “the suppression of potential,” “denial and abandonment by family,” “poverty and disability,” “expansion of thinking,” and “opportunities of special participation”; and (3) the positive reinforcement process: “self-disclosure and jump-starting life,” “maintain satisfaction and achievement,” and “socioeconomic independence.” These findings expand the understanding of the factors common to the coping process experienced by individuals with visual impairment and highlight the importance of psychological support, family, education, and social support.

## 1. Introduction

The number of people with visual impairment caused by environmental and physiological changes has increased in recent years. People with visual impairments have a variety of difficulties associated with their development and life activity along with physical discomfort. In particular, they are exposed to diverse and complex environments while coping with physical development, mental depression, frustration, and social adaptation [1]. Furthermore, children with visual impairments experience numerous barriers in their development, physical activity participation, and the whole education curriculum, from early life through adulthood [2].

Young children with visual impairments also experience a variety of problems that can lead to emotional isolation, loneliness, alienation, and frustration, as well as physical difficulties in daily activities [1]. Moreover, there is a lack of educational opportunities to promote healthy human development end reduce isolation in the social environment, and no opportunity to gain important knowledge and positive information [2].

Despite these challenges, factors that can positively affect developmental processes, such as coping on family support, social environment, and children’s ability to adapt, can have a positive influence on children with visual impairments [3]. One way to help visually impaired people overcome the impediments of their disability is to strengthen their adaptation to life. Adaptation has been defined as the capacity to transcend adversities and the necessary social skills to bring about positive results [4]. Craig [5] has demonstrated that coping provides protection against the negative consequences of having a disability, suggesting the presence of a relationship between coping and overall outcomes in children with disabilities.

In research on the coping process of people with visual impairments, it is important to focus on identifying factors that lead to positive results by clarifying the relationship between stressful situations and individual disabilities [6]. However, existing intervention studies that have focused on facilitating coping using positive adaptation through in-depth discovery and the analysis of empirical factors in the developmental processes of people with visual impairments are insufficient [7]. Moreover, the study was limited to studies focusing on the individual’s adaptation to adversity, and the study considering the means to improve social adaptation needs to be considered first.

In order to grasp the factors that can improve the quality of life in the social adjustment process for people with visual impairments, research is needed to clearly identify the problems they are experiencing and what exists in the coping process [8]. If these specific intervention goals are properly met, the quality of life of people with visual impairments could be significantly improved.

This study aims to analyze the difficulties and crises of living with visual impairment, positive coping factors, and support system environment to help overcome them. In order to analyze the factors existing in the coping process, it is necessary to first understand the positive support system and environment that exist based on physical, mental, and social experiences in the life process from infancy to adult development.

In addition, there is a need to understand the support systems that positively affect parents, siblings, economics, education, and social environments that are included in the internal and external environments that influence growth and development. Therefore, this study intends to analyze the factors included in the positive coping process by specifically analyzing the empirical life of a person with visual impairment from childhood to adulthood.

The purpose of this study is to understand the factors of coping and contribute to the development of coping programs for people with visual impairments, based on the description of the coping factors that can enable the positive developmental process of those. In order to understand the process of coping life and related factors, the research questions were as follows.

- What kind of experience have you faced as a result of your visual impairments based on a retrospective perspective of your life?
- What are the positive factors that have influenced life, and how do they affect the process of coping?

## 2. Materials and Methods

A qualitative research method that used an interpretative phenomenological analysis (IPA) was employed to explore and interpret the meaning of the complexity experience of the whole life story of children and adults with visual impairment. Therefore, this study needs to do an in-depth analysis by narrowing the scope of the participant’s development process by applying IPA. The IPA research method is use to explore and make sense of an individual’s perception or explanation of health, disease, protection, relationships, emotions, and coping processes [9].

IPA consists of three key elements: phenomenology, hermeneutics, and idiography. The phenomenological element includes a detailed examination of the participant’s world of life, and hermeneutics is an in-depth two-step process of interpretation that refers to the process of making the participant’s meaning more detailed through dual analysis [10]. Finally, on the basis of this is an idiography. Therefore, when these three elements are complemented and performed properly, researchers can approach the participant’s experiences and pay attention to detail [11].

IPA allows researchers to describe the individual’s experiences through their life story [12]. This study used IPA to attempt to identify the meaning in and the interpretation of the participant’s experiences with the process of coping. Further, over the course of analyzing their experience in this study, researchers tried to maintain curiosity and interest in the participants’ life story on the chronology of events through flexible and open interviews. The stages of the research process are divided into collecting data, constructing the interview schedule, interviewing, analysis, and writing up [10].

### 2.1. Participants

Eight persons with visual impairment who lived in Seoul, South Korea were recruited through purposive sampling. The aim of researchers using IPA was to gain a detailed account of individual experiences to foster a deep understanding of the phenomenon. Participants were recruited over the phone through disability organizations. The researcher explained the study’s purpose and procedure, and selected the participants who wished to volunteer for the interview. The inclusion criteria required the participants to:

- Visual impairment from childhood.
- Be unable to recognize objects at close range such as severe visual impairment and blindness.
- Be able to communicate with others.
- Have graduated with a bachelor’s degree or higher.
- Have professional work experience.

Participants were required to understand the purpose of this research and to be able to provide informed consent. Their ages ranged from 50 to 60 years. Participants included 2 men and 6 women who are visually impaired (Table 1). The participants were targeted at those who fully understood the process of this interview and were able to explain the experiences of the happy and difficult moments in their own lives.

### 2.2. Ethical Considerations

This study was conducted after receiving approval from the Chung-Ang University institutional review board (1041078-201312-HR-00109-03). The participants were informed about the study and could withdraw their participation at any time. There was no disadvantage for those who did not participate in the study. Personal identification information was encrypted and statistically processed to protect the privacy and confidentiality of the participants. In the process of talking about past experiences, when the participants were emotionally struggling and weeping, the researcher paused and sympathized, waited until the mind was relaxed, and resumed the interview. All procedures in this study were performed in accordance with the ethical standards of the institutional and/or national research committee, and with the 1964 Helsinki declaration and its later amendments or comparable ethical standards. Informed consent was obtained from all participants included in this study.

### 2.3. Data Collection

For data collection, in-depth interviews were conducted three to four times each from 2016 to 2018 using semi-structured questions, and the author with research assistants participated in the entire interview process directly to collect relevant information. After receiving the permission to record, participants’ interviews were recorded using a tape recorder, and in a notebook if necessary. Each interview utilized an unstructured interview method and was conducted several times to collect the information more precisely, and each discussion lasted approximately 60–80 min. Participants were asked to candidly discuss their experiences of their whole life. They were asked to provide a full description of their experiences and to tell a variety of stories in order to convey their perceptions of the bearing of education and the social and cultural backgrounds of their life.

The participants were asked to tell their whole life stories once they were relaxed and in a comfortable position. The interviewers asked them to provide their thoughts about their experiences regarding visual impairment, in addition to relaying the story’s events. Further, they were asked to describe what they thought the implications were regarding the event they were describing, along with a specific interpretation of those events. Finally, the participants were asked to narrate a variety of stories so that their social and cultural backgrounds as individuals with visual impairment could be obtained.

### 2.4. Data Analysis

The study analysis consisted of repetitive and induction cycles. Analyzing the entire recording of the interview resulted in a meaningful and common expression [8]. In the process, an understanding of the participants’ experiences appeared, and finally, a theme was completed that could be interpreted to solve the research problem. The final analysis was categorized according to different aspects of the coping process.

The analysis was divided into five stages; the first step was revisiting and reading the data set. This step reads the data collection and reviews it. Documenting along with recording the recorded content clarifies what the researcher thinks at each stage of the analysis and increases reliability. The interview was conducted through retrospective contents from childhood to becoming a member of society. Then, the participants listened to the recordings of the stories that they shared. During the analysis phase, various information was added to include the participant’s personality, experience, history, and social situation.

An audio recording of each participant’s narrative was prepared. During its preparation, the researchers removed any repeated personal opinions. The focus of this stage of analysis was to interpret the information in the narrative with regard to the participant’s coping. Thus, each story was examined for instances of coping, defined as ways in which the participants overcame difficult situations in childhood and how they were able to adapt to society.

In the second step, the initial themes explaining the nature of the experience were identified. Then, the data and records analyzed in meaningful units were transformed into a new language, the relationships between the themes were identified and ensured the hermeneutic principles of revising with further conversation. The fourth step was to repeat the data analysis process through subsequent conversations. Lastly, in order to maintain the reliability of the measurements during this process, the authors read together what was recorded with qualitative research professors and other experts, collected common interpretations, and clustered them to derive the final superordinate themes. Finally, to confirm that these derived themes matched the participants’ experiences, the authors checked whether their experiences match those of other visually impaired subjects who did not participate in this study.

## 3. Results

This study explored the process of coping with visual impairment in the growth process of life. To this end, in-depth interviews were conducted on research participants and data were collected and analyzed using phenomenological qualitative research methods. The result of this study was to classify the process of coping into three themes of the coping processes and to derive eleven components including both superordinate and subcomponent themes. Therefore, this study analyzed the meaning and process to coping in the life process of people with visual impairment. The results of three superordinate themes of the identified coping processes were: experience and adaptation, circumstance, and reinforcement. The experience and adaptation process included the themes “lack of awareness of having a disability” and “effect of the environment on self-awareness.” The circumstance process contained the themes “the exposure to concealment and abuse,” “the suppression of potential,” “denial and abandonment by parents,” “poverty and disability,” “exchange and self-regulation,” and “social integration.” The final superordinate theme was the reinforcement process, and it contained the themes “self-disclosure and jump-starting life,” “maintenance of a positive attitude,” and “social self-reliance” (Table 2).

### 3.1. Self-Awareness and Adaptation Process

Participants began to remember their positive or negative experiences and the process of adaptation, usually when they heard and recognized information about their disability and its causes. As a result, recognizing that they had a disability, they were able to understand and adapt their environment and situation.

#### 3.1.1. Self-Awareness of Disability

At the beginning of life, it is difficult to recognize visual impairment. The results of this study showed that although it was difficult for participants to develop their identity as visually impaired people, after recognition, various factors contributed to having positive motivation in life. In addition, it explains how that helps to realize the process of growth regarding visual impairments in interactions with others.

My parents told me when I was 5-years-old that I was blind because of a chicken pox infection. I did not know how I was blind until then, it was just hard to see and I was aware of this situation. (# 1)

One day when my sister asked me the color of the cloth she was holding in front of me, I could not say it. At that time, I slowly realized that I could not see. But I was so young that the negative feelings about these processes and events was not very strong and I gradually got used to them. (# 4)

#### 3.1.2. Adaptation to Disability and the Environment

When participants first learned about their disorder, it was through their family and friends. As children, participants learned how to adapt to their disability through trial and error, and through interactions with their non-disabled peers.

I did not blame people for my disability. I played well with friends. I was just like that in my childhood, but there were many other disabled children who were isolated from society. (# 8)

My father was a teacher. My parents did not want me to go to school. So, I was with my brothers and sisters. When they went back to school, my parents taught me to sing a song, and we went to a school classroom to play the piano and sing. I loved songs and my dream was to be a singer. (# 7)

When participants first recognized their impairment, they reported this as not being problematic. They spent time with their siblings and other non-disabled children, and learned how to interact with others through communication and a variety of shared activities. This experience enabled them to build the basic skills necessary for being active members of society.

The hardship was that I fell into a pond when I ran in the fields and mountains with my friends. I floundered in the water, and suddenly my grandfather grabbed my hands, and I was alive. I never thought too much about that event at that time because I was too young. However, now I think that if those friends had prevented me from going there (laughter), I would not have fallen in the water. I do not know; I just ran way too fast without time for them to say, “be careful.” (# 2)

Though the participants in this study described problems when navigating their environment, they still spent time with friends who provided them opportunities to ask questions and develop methods of better navigating their environment, which resulted in positive experiences. Moreover, participants felt that their quality of life had not been reduced compared to people without disabilities.

### 3.2. Facing the Circumstances Process

The second theme of this study are the steps facing different situations. They had difficulties such as concealment and abuse from their families and siblings, and despite their potential, there were fewer opportunities to receive education due to economic difficulties and disabilities. Nevertheless, they learned how to expand their thoughts and actions in the face of this difficult situation, and involve dealing with relationships with other members of society who share the same self-problem.

#### 3.2.1. The Exposure to Concealment and Abuse

Most participants became aware of visual impairment at the early stages of school age, and since then began to feel alienated from their families, and as they grew up, they experienced more and more social prejudice. They blamed others for their inattention, the lack of familial support, and their feelings of sorrow and guilt owing to their disability. In some cases, participants appeared nervous, hands trembling and faces rigid, when recalling behaviors that involved abuse by a family member.

Although my mother was well-educated, she hurt me because our family was poor. I resented my parents because I was blind, and they could not manage me. It was not my fault… but, why did she mistreat and hate me? (# 5)

Participants often appeared angry or cried when they recalled these traumatic events. Although the process of coping was derived from their resentment toward family, recalling the past was very difficult for participants. They described themselves as being self-made as a result of their experiences, and regretted the blame they experienced from their family.

#### 3.2.2. The Suppression of Potential

Participants expressed strong emotion when recalling people’s ignorance regarding their individual potential because of their disability. They felt this was the most difficult and unfair experience during both familial and communal interactions.

Disabled children have some things that they can do well, such as write characters, sing a song, or play the piano. They can do something well. (# 1)

Visually disabled children have a simple disability in some circumstances, but their parents will not leave them alone. They do not allow them to do anything by themselves. (# 4)

These experiences highlight the importance of identifying the potential abilities of children with disabilities and of providing them with opportunities to develop their skills.

#### 3.2.3. Denial and Abandonment by Family

Participants and their parents were affected by the negative social and institutional emphasis placed on individuals with disabilities. In particular, an attitude that the participants should accept continuous abuse, fear of social scrutiny, and feelings of helplessness arose as a result of negative views on and a lack of knowledge about visual impairment, as well as economic disparities. Commonly, participants recalled feelings of resentment and hurt, and reported that their parents had acted negatively toward them.

My mother didn’t like me to appear before them when someone came home. And when my family had to go to a special place, I left me alone. When I felt this kind of discrimination, I was so stressed and hard, and I wanted to live alone. (# 5)

#### 3.2.4. Poverty and Disability

Most participants did not come from affluent families and had to obtain jobs to earn money rather than pursue an education. This negatively impacted their ability to communicate due to the lack of educational opportunities, and it increased their isolation.

When I was seven, I had to learn how to earn money. … to learn braille and further my studies. …but they wanted me to earn money to support the household rather than to study. (# 3)

I wanted to go to school normally with my sisters and brothers. By the way... it was hard to attend formal school with special disabilities... (# 6)

The thing that was hard about school was that my parents had no money. When I needed to print braille on high-quality paper, I had no money to buy the paper. So, I borrowed paper from friends. (# 5)

#### 3.2.5. Expansion of Thinking

Although the participants experienced economic, familial, and societal hardships, some circumstances led to gratitude, enabling them to adapt to difficulties. Advice from a visually impaired senior played an essential role in providing guidance to participants.

There is a community for the visually disabled. In it, people help each other. It is a very helpful and essential aspect of any situation. (# 1)

He was also blind. He told me that there was a school for visually impaired people. They taught writing, reading, and printing in braille. I thought that if I could learn braille, I would be able to study more. (# 4)

Participants experienced the ability to expand their thinking, not only through family and siblings, but also through meetings with other visually impaired people in the process of growth and through the exchange of information between their communities.

#### 3.2.6. Opportunities of Special Participation

People with visual impairments eventually received help from the community of people with the same disabilities to assist with solving problems, and there was an experience of being comforted by and integrated into the environment.

During summer vacation, I stayed alone in a boarding school for the disabled. Nobody from my family came to pick me up. One day, a friend who could see a little more than me took me to my house. Eventually, a child in the same position helped me... Of course, the family wouldn’t have liked my coming. (# 6)

When I studied in a disability special school program, they helped me write a report and recorded the lecture. They really understood my situation. The teacher treated me as a special person and, after class, rewarded me in front of other students. I was proud and proud of myself for the first time. (# 4)

### 3.3. Positive Reinforcement Process

The final stage of the coping process experienced by the visually impaired was named positive reinforcement. In this process, a situation was devised that overcomes existing difficulties and opportunities and begins with challenges and new forms of life. The attitude of life had been strengthened positively, and the process includes having the independent ability to overcome the conditions, adapted to society by themselves, and even strengthen economic abilities.

#### 3.3.1. Self-Disclosure and Jump-Starting Life

Participants had to run out of the world to live like others, and tried to learn what they wanted to do and learn other skills. In addition, they gradually learned how to understand and adapt to the world, and they chose the way to jump and create something to live a better life.

I wanted to study and live with other normal people, but I didn’t. My parents didn’t have enough money and didn’t study enough so I wanted to study… One day I took my bag out of my house and ran to the train station …It was a new challenge and beginning in my life. (# 7)

When my father asked me if I wanted to study, I answered firmly. I wanted to go out and study too much. My thought was that I wanted to learn the same way as other people, and I had to prepare for social life. I couldn’t really understand why I couldn’t do it just because I had a disability… (# 1)

First, I learned braille, and then I learned some techniques to handle a machine. During that time, I invented a machine to make a rope. My teacher saw the machine and was surprised at how nice it was. So, I left a brilliant thing for the school that can be used to help other students. (# 8)

In the hotel, many Japanese people would come for a massage. At that time, it was popular to give massages to foreigners. So, I learned Japanese, and I was good at speaking it. I could talk to the people, they liked me, and I earned a lot of money. (# 1)

#### 3.3.2. Maintain Satisfaction and Achievement

Participants were able to positively cope in society and made enterprising efforts to maintain a positive attitude despite feelings of loneliness and pain.

When I entered society, I decided I needed to succeed and make money as if I did not have a disability. It was hard for me to go anywhere and to study, but I have tried diligently. If I get the opportunity, I want to be a professor at my school. (# 1)

The greatest achievement that I have felt is experiencing my children’s weddings. Moreover, I was very happy to achieve a certificate in social welfare. I have been a sponsor for World Visions for five years. I am very pleased that I can help others. (# 5)

By putting aside all their childhood difficulties, participants were able to identify methods to live constructively and independently as active and normal adults despite visual impairment.

#### 3.3.3. Socioeconomic Independence

Participants’ enhanced social involvement became an experience in overcoming difficult childhood situations and expanding opportunities for social activities. Participants explained that their attitudes were increasingly strengthened to increase social independence. Individuals with visual impairments have limited career choices and are not fully autonomous, but have been able to create professional jobs by trying to do what they can. As a result, social and economic independence have been achieved.

What I was able to do independently in society was performing massage, acupuncture, working as a teacher for disabled individuals and manager in disabled centers. This professional work required a lot of personal effort. I was lucky though. I’m studying and working as a manager in society …In addition to these jobs, people with disabilities need to develop more diverse jobs. I will work to develop these different careers. (# 7)

Because people without disabilities have more career opportunities than we do, we must secure what only blind people can do. For example, we need to be supported by society so that we can monopolize the task of doing massage. We are good at massages, so society needs to secure the environment for us to act and work. (# 5)

## 4. Discussion

As a result of this study, the experiences of the coping process that existed in the course of life from childhood to adulthood of the visually impaired were identified as three main themes such as self-awareness and adaptation process, facing the circumstance process, positive reinforcement process. These processes were experienced in the early childhood including preschool age, school age, adolescence, which could affect a person’s attitude formation to adult stages of life through the perception of their disability, discomfort experienced, and the process of adapting themselves slowly. In this section, we tried to explain and interpret the three analyzed themes in detail.

### 4.1. Self-Awareness of Disability and Adaptationin

The first experience in recognizing a disability in childhood involves psychological, physical, or spiritual difficulties. Children who are aware of difficulties develop an adaptation stage using both negative and positive factors. Participants in this study also experienced negative factors and frustrations when they first perceived their disability, and they began to adapt to different needs, which required basic effort.

Kumpfer [13] suggested that both external and internal variables can mediate an individual’s acceptance of both positive and negative factors. According to Extremera and Rey [14], coping is a process of adaptation, and children who do not adapt may face psychological and physical illness because of negative and traumatic experiences.

Negative experiences can reduce the likelihood of participants successfully adapting to life with blindness. However, Kumpfer [13] explained that an individual can adapt, given the individual and psychological or social environmental factors involved, regardless of whether the child’s experience is positive or negative. In this study, the similarities between experience and adaptation categories constituted the concepts of awareness of disability, adaptation to disability, and environment. It was not easy to recognize children’s visual impairment for the first time, but it showed that the children were motivated to shape their identity according to their environmental factors.

The memory of awareness of disability and its adverse effects are directly influenced by the integrated environment, which can be explained as the basic step toward adaptation, affecting the first stage of recognition and adaptation to childhood disorders. Participants explained how their interactions with others helped to realize the presence of visual impairment. It was through experiences with one’s family and friends that one became aware of being a child with a disability and being different from others.

Recognizing this, the participants learned how to adapt their interactions with family and non-disabled peers through various trials and errors. They spent time with their siblings and other children without disabilities, learned how to interact with others through communication and various sharing activities, and through these experiences they gained the basic skills needed to cope with life’s challenges as visually impaired people are more likely to be exposed to more dangerous situations than non-disabled people [4]. However, the positive attitude presented by the participants accepts risks and inconveniences and adapts to the environment. The people involved in this study explained that they had trouble exploring their environment, but still spent time with their friends asking questions and developing a better way of exploring their environment to gain a positive experience. In addition, participants thought that the quality of life did not decline compared to those without disabilities.

### 4.2. Facing the Circumstances Process

In this study, the second stage of adaptation experienced by those with visual impairments through adaptation appeared to be the process of adapting to various types of situations during the growth process.

Coping with the situations you face in your life can help people to accept and adapt to positive or negative circumstances. Researchers have reported that coping can be improved by creating opportunities to overcome adverse conditions and reinforce positive situations [15,16]. Watson [17] said that children who can establish an identity try to keep the control of disability and rebuild, understand their lives, and have a positive attitude.

#### 4.2.1. Exposure to Concealment and Abuse

Participants realized that they were disabled as a child, and experienced not only loneliness and alienation through concealing themselves from their families, but also many prejudices from the social environment while growing up. People with disabilities are said to have more sadness and guilt when their family lacks support or is dissatisfied and alienated [18]. When these emotional problems persist for people with disabilities, it is a factor that can cause various problems in the growth process, so families must understand the difficulties of children with disabilities and support them without prejudice. In addition, social environment resources that can utilize support programs and social support systems should be prepared so that families can overcome stress and fatigue caused by responsibility.

#### 4.2.2. Suppress Potential Ability

Even if they have a disability, they need to find and develop not only the thinking and creative thinking that exists for them, but also other potential abilities beyond the disability. If you have a disability in one part, developing potential skills in the other part can compensate for the lacking and lead a balanced life. In particular, if people with disabilities can develop and strengthen their potential abilities, they can gain self-confidence and effectively cope in the disability process. Prior studies have also emphasized the importance of identifying the potential abilities of children with disabilities and providing them with opportunities to develop skills [17,18].

#### 4.2.3. Denial and Abandonment of the Family

Participants and their parents were affected by negative social and institutional emphasis on individuals with disabilities. In particular, participants’ continued abuse, fear of social investigations, and attitudes toward helplessness resulted from negative views on visual impairments and lack of knowledge about visual impairments and economic deficits. In the results of this study, not only did participants feel anger and hurt due to the negative view of society, parents reported that they also showed negative behavior to participants. To help parents support their children by reducing the stress, conflict, and anxiety associated with these disabilities, it is necessary to strengthen parental self-esteem and receive early education [19]. Otherwise, the participants would experience a lack of developmental growth, and their ability to become an independent member of the community was hindered. Speedwell et al. [20] reported that over 80% of parents of children with visual impairments had no access to parenting and educational resources prior to diagnosis. Participants and parents experienced unnecessary stress when they learned through “trials and mistakes” [21]. Parental education regarding the needs of children with visual impairments should occur as soon as possible to prevent conflict and anxiety and to encourage the process of developing coping.

#### 4.2.4. Poverty and Disability

Most participants grew up in low-income families and had to get a job to make money from an early age instead of being educated. This negatively impacted communication skills due to the lack of educational opportunities and left them isolated. There is also a higher cost of education for children with disabilities, which increases problems [22]. Bakker, Steultjens, and Price [23] reported that children with visual impairments in high-income families experience rich educational and cultural support, while children in low-income families do not have the same opportunity.

#### 4.2.5. Exchange and Self-Regulation

The participants in this study suffered from economic, family, and social difficulties; despite this, many were able to adapt to the difficulties. In particular, the advice of other adults with visual impairments played a crucial role in guiding participants. The exchange of information between participants, the elderly with visual impairments, and the visually impaired community played an important role in the participants’ lives. The elderly and visually impaired people, in particular, knew what the participants’ actual needs were and provided support by improving their coping skills, independence, and self-confidence by providing advice on issues related to intellectual and physical disabilities [24]. As Maxey and Beckert [25] pointed out, these opportunities provided participants with a sense of accomplishment related to the development of their identity and confidence. A high level of awareness of the needs of people with disabilities plays an important role in providing opportunities for the visually impaired.

#### 4.2.6. Social Integration

Participants’ socioeconomic independence stage is the last sub-theme. The process of coping with being visually impaired from a difficult childhood to adulthood not only strengthens the capacity for social participation but also provides independent economic capabilities [26]. Participants experienced the process of accomplishment. In the process of socioeconomic independence, it was found that social environment, the social atmosphere that enables the disabled to participate, and environmental factors must be strengthened. Additionally, providing social support and attention that can expand their occupational opportunities is needed.

### 4.3. Positive Reinforcement Process

The last concept common to the visually impaired is the process of positive self-reinforcement. The subthemes are self-disclosure and jump-starting life, maintenance of a positive attitude, and socioeconomic independence. The power of the positive reinforcement process enhances self-esteem and reinforces positive thinking despite disabilities. These results show that they can improve their abilities and maintain their expertise in each field.

According to Masten [27], coping is a process that integrates social, psychological, and family elements into a protective mechanism that is evident throughout an individual’s life. Participants were able to complete the necessary daily tasks through positive reinforcement, which helped them to achieve independence. The combination of processes that result in self-fulfillment and happiness can become a driving force in people’s lives [27,28]; these assist blind individuals in achieving their rights as community members.

The participants’ longing for the type of life lived by those without a disability was strong despite their poor economic background and parents’ lack of knowledge. Thus, this became a driving force in participants’ efforts to improve their situation.

Gibson, King, Teachman, Mistry, and Hamdani [29] stated that constraints in the physical environment often force disabled people to be passive observers rather than active players in daily activities. However, the participants in this study actively challenged the negative forces in their lives, not allowing their visual impairments to limit their development of individual skills. Through education, participants were able to discover and utilize their talents in society.

Education gave the participants opportunities that would have been impossible otherwise. According to Sansom [30], children with disabilities who do not receive education do not discover their talents and have a greater chance of failure.

Though struggle was inevitable for the visually impaired participants, the prejudice of community, absence of family support, and instances of abuse and concealment by family members also served as an opportunity to expand their capacity for self-reinforcement and self-expression. Participants were able to realize through these experiences that a visually disabled person can live independently in the community.

In addition, appropriate education played a large role in their ability to adapt and develop coping skills. Moreover, the discovery and development of their individual talents further enhanced their social adaptation and self-reliance. Participants were able to positively cope in society and made enterprising efforts to live positive lives despite feelings of loneliness and pain. By putting aside all their childhood difficulties, participants were able to identify methods to live constructively and independently as active and normal adults despite their visual impairment.

As such, the findings of this study indicate that there are common stages of adaptive ability, coping with situations, and self-reinforcement among people with visual impairment. It is considered a very meaningful process to reconfirm through future research.

## 5. Conclusions

The main purpose of this study was to analyze and understand the coping processes that exist in the growth process of life with visual impairment and what factors influence coping processes. The second purpose of the results was to provide useful rationale and data for people with visual impairments and their families.

The study analyzed the development of children with visual impairments through stories describing childhood experiences. Stories on eight topics were categorized into three processes: experience and adaptation, the circumstances process, and positive reinforcement process. The characteristics of coping children with disabilities are seen in the educational environment and social integration associated with peers and families. Characteristics that enhance coping skill include young children’s self-awareness and adaptation, facing the circumstance, positive reinforcement process. Even when living with a visual impairment, interventions can be designed to enhance the basic characteristics of coping and enrich the educational environment and encourage social attention to increase positive coping. To do this, it is also necessary to strengthen the intervention and capacity with families, to present specific measures and supportive social programs, and to utilize available resources to improve the development of children with visual impairments. The most important factors like this are high self-efficacy, social support, social skills, and professionalism. In order to strengthen and activate these elements, the development of a program system that can be applied to children with visual impairments and their families is required.

This study aimed to highlight the prejudices against disabilities and to emphasize love, consideration, and sympathy for others. The aim should be to reduce prejudice; as such, interventions are needed to increase understanding and encourage acceptance.

## Figures and Tables

**Table 1:** Characteristics of the participants.

Name	Sex	Age	Onset	Status of Visual Impairment (Presenting Distance Visual Acuity)
Choi	Female	58	Complications caused by infectious diseases	Severe visual impairment Worse than: 1/20 (0.05)
Lee 1	Male	60	Complications caused by infectious diseases	Blindness Worse than: 1/50 (0.02)
Lee 2	Female	58	Congenital	Blindness Worse than: 1/50 (0.02)
Jung	Female	56	Congenital	Blindness Worse than: 1/50 (0.02)
Kim 1	Female	60	Complications caused by infectious diseases	Blindness Worse than: 1/50 (0.02)
Kim 2	Male	56	Acquired by accident	Severe visual impairment Worse than: 1/20 (0.05)
Park 1	Female	50	Congenital	Blindness Worse than: 1/50 (0.02)
Shim	Female	57	Acquired by accident	Severe visual impairment Worse than: 1/20 (0.05)

**Table 2:** Superordinate themes of the coping processes.

Superordinate Theme	Subthemes
Superordinate Theme 1: Self-awareness and adaptation process	- Self-awareness of disability - Adaptation to disability and the environment
Superordinate Theme 2: Facing the circumstance process	- The exposure to concealment and abuse - The suppression of potential - Denial and abandonment by family - Poverty and disability - Expansion of thinking - Opportunities of special participation
Superordinate Theme 3: Positive reinforcement process	- Self-disclosure and jump-starting life - Maintain satisfaction and achievement - Socioeconomic independence
